# HPGDS is a novel prognostic marker associated with lipid metabolism and aggressiveness in lung adenocarcinoma

**DOI:** 10.3389/fonc.2022.894485

**Published:** 2022-10-17

**Authors:** Fengling Shao, Huajie Mao, Tengling Luo, Qijun Li, Lei Xu, Yajun Xie

**Affiliations:** ^1^ The Ministry of Education Key Laboratory of Laboratory Medical Diagnostics, The College of Laboratory Medicine, Chongqing Medical University, Chongqing, China; ^2^ Department of Laboratory Medicine, The First Hospital of Xi’an, Xi’an, China; ^3^ Laboratory Animal Center, Chongqing Medical University, Chongqing, China

**Keywords:** hPGDS, lipid metabolism, lung adenocarcinoma, biomarkers, bioinformatics

## Abstract

**Background:**

Lung adenocarcinoma (LUAD) is the most common respiratory globallywith a poor prognosis. Lipid metabolism is extremely important for the occurrence and development of cancer. However, the role of genes involved in lipid metabolism in LUAD development is unclear. We aimed to identify the abnormal lipid metabolism pathway of LUAD, construct a novel prognostic model of LUAD, and discover novel biomarkers involved in lipid metabolism in LUAD.

**Methods:**

Based on differentially expressed genes involved in lipid metabolism in LUAD samples from The Cancer Genome Atlas (TCGA), abnormal lipid metabolism pathways in LUAD were analyzed. The lasso penalized regression analysis was performed on the TCGA cohort (training set) to construct a risk score formula. The predictive ability of the risk score was validated in the Gene Expression Omnibus (GEO) dataset (validation set) using Kaplan-Meier analysis and ROC curves. Finally, based on CRISPR gene editing technology, hematopoietic prostaglandin D synthase (HPGDS) was knocked out in A549 cell lines, the changes in lipid metabolism-related markers were detected by western blotting, and the changes in cell migration were detected by transwell assay.

**Results:**

Based on the differential genes between lung cancer tissue and normal tissue, we found that the arachidonic acid metabolism pathway is an abnormal lipid metabolism pathway in both lung adenocarcinoma and lung squamous cell carcinoma. Based on the sample information of TCGA and abnormally expressed lipid metabolism-related genes, a 9-gene prognostic risk score was successfully constructed and validated in the GEO dataset. Finally, we found that knockdown of HPGDS in A549 cell lines promoted lipid synthesis and is more invasive than in control cells. Rescue assays showed that ACSL1 knockdown reversed the pro-migration effects of HPGDS knockdown. The knockdown of HPGDS promoted migration response by upregulating the expression of the lipid metabolism key enzymes ACSL1 and ACC.

**Conclusion:**

The genes involved in lipid metabolism are associated with the occurrence and development of LUAD. HPGDS can be a therapeutic target of a potential lipid metabolism pathway in LUAD, and the therapeutic target of lipid metabolism genes in LUAD should be studied further.

## Introduction

Lung cancer is the most common cancer in the world (1), and its incidence is increasing every year. It is the leading cause of cancer-related mortality, making it a major global health problem (2). Adenocarcinoma is the most common histological subtype of lung cancer in both men and women (3). Even although the development of treatment strategies and new drug discoveries in recent years have resulted in prolonged survival of lung adenocarcinoma (LUAD).However, its five-year survival rate is only 15%. Recent studies have suggest that more than half of patients have missed the targetable gene alterations period that can improve their survival rate (4). Therefore, the discovery of specific early detection markers and therapeutic targets is the key to improving the survival rate of patients with LUAD (5, 6).

Lipid metabolism, including uptake, storage, and lipogenesis, occurs in various types of cancers, such as pancreatic, hepatic, and colorectal cancer, and affects tumor resistance and therapeutic efficacy (7–10). Previous studies on lipid metabolism showed that patients with higher lung cancer having high levels of high-density lipoprotein cholesterol (HDL-C), low-density lipoprotein (LDL), and low-density lipoprotein receptor (LDLR) have better survival rates (11, 12). Compared with the control group, the levels of some lipid metabolism-related products in the serum of patients with nonsmall-cell lung cancer (NSCLC) were significantly increased (13). The activity of cancer cells is accompanied by a large consumption of ATP, and fatty acid oxidation (FAO) can help generate ATP by coordinating the activation of lipid anabolism (14). Therefore, elucidating the underlying lipid metabolism-related mechanisms of LUAD will help to increase clinical treatment and thus prolong the survival of patients.

Hematopoietic prostaglandin D synthase (HPGDS, an enzyme that produces prostaglandin D2) is a σ class glutathione transferase, which was discovered 40 years ago (15–17)HPGDS is involved in the arachidonic acid metabolic pathway, and catalyzes the production of prostaglandin D2 (PGD2) (18). HPGDS is associated with some pulmonary inflammatory diseases (19, 20). It is also associated with pancreatic tumors and testicular germ cell tumors (21, 22). HPGDS plays an important role in the occurrence and development of lung cancer, however, its catalytic product, PGD2, has been confirmed to be a mast cell-derived anti-angiogenic factor for lung cancer (23). Therefore, the relationship between HPGDS and lung cancer should be further revealed.

In this study, we used bioinformatics to analyze the characteristics of lipid metabolism-related genes in lung cancer, and found differential lipid metabolism pathways between lung cancer and normal tissues. Risk signatures of lipid metabolism-related genes were established and validated in external datasets. In the process, the recurring appearance of HPGDS was intriguing. Finally, we performed cell biology experiments to demonstrate that the knockdown of HPGDS promoted adipogenesis and increased the migration of lung cancer cells.

## Materials and methods

### Datasets and genes involved in lipid metabolism

LUAD and lung squamous cell carcinoma (LUSC) gene expression patterns and clinical data were collected from The Cancer Genome Atlas (TCGA), wherein the LUAD dataset included 516 tumor samples and 59 paracancerous tissues, while the LUSC dataset included 501 tumor samples and 108 paracancerous tissues. The Gene Expression Omnibus (GEO) was searched for microdata on the mRNA expression. GSE72094 is a LUAD dataset composed of 442 samples, based on the GPL15048 platform, for external validation of the risk score. GSE74777 is a LUSC dataset based on the GPL17586P platform, containing 107 samples, which were used as an external validation for LUSC. Based on the method described by of Deng et al, we downloaded the lipid metabolism-related genes from the Molecular Signature Database (version 7.0) (24).

### Screening and functional enrichment of lipid metabolism-related differential genes

R “Limma” (R.4.1.0) was applied to identify differentially expressed genes (DEGs) between tumor tissue and normal tissue with a false discovery rate FDR <0.05 and |log2FC| ≥2 for the assessment of the involvement of significantly different genes in lipid metabolism. Functional enrichment analysis of GO and KEGG was performed using by DAVID (V.6.8 https://david.ncifcrf.gov/tools.jsp).

### Protein-protein interaction network and subcluster analysis

The PPI of DEGs was predicted using the STRING online platform (http://string-db.org/). Hub genes were calculated with reference to the “Degree” algorithm in the cytohub plugin in Cytoscape (v.3.8.2) and the visualization was calculated.

### Construction and validation of the prognostic risk score

Based on the lasso algorithm, the prognostic risk scoring formula was constructed with the TCGA dataset as the training set, and the GSE72094 and GSE74777 datasets were used as the external validation datasets for LUAD and LUSC, respectively. Independent prognostic factors were identified by multivariate Cox regression analysis using the survival R package. The patients were assigned to the high- and low-risk groups in accordance the median risk score, and Kaplan-Meier analysis was performed to plot overall survival (OS) and ROC curves in order to assess the prognostic power of the risk score.

### Gene set enrichment analysis

To explore the underlying molecular mechanisms of prognostic risk score and HPGDS, we applied the Gene Set Enrichment Analysis(GSEA)of the underlying molecular mechanisms (25, 26). C2, C5, and C6 were searched to identify the oncogenic signatures of KEGG pathways, biological processes, cellular components, molecular functions and dysregulation(P < 0.01,FDR < 0.05).

### Plasmid construction

The 20-nt target DNA sequences preceding a 5,-NGG PAM sequence in the genomic HPGDS and ACSL1 were selected for generating a single guide RNA (sgRNA) for the SpCas9 targets by using the CRISPR design website (https://design.synthego.com/#/). *Homo sapiens* HPGDS and ACSL1 sgRNA were cloned into the lentiCRISPR v2 vector at the site of BsmBI with sgRNA forward CACCGGCCTCATCTTATGCAAGACT and reverse AAACAGTCTTGCATAAGATGAGGCC, forward CACCGGAAGAGTACGCACGTACTGT and reverse AAACACAGTACGTGCGTACTCTTCC. Definitively, to confirm the DNA sequence, the DNA was successfully cloned, after which the plasmid was sequenced and aligned (BGI, Chongqing, China).

### Cell culture and transfection

A549 (human non-small cell lung cancer line) was sourced from the American Type Culture Collection (ATCC) and cultured in Dulbecco’s Modified Eagle Medium (DMEM) (Gibco, Carlsbad, CA, USA) supplemented with 10% fetal bovine serum (FBS, vol/vol, Biological Industries) and 1% penicillin/streptomycin (Invitrogen, Grand Island, NY, USA). The cell line was cultured at 37°C under a 5% CO_2_ atmosphere. sg-HPGDS, sg-negative control (sg-Con), and sg-ACSL1 plasmids were transfected into A549 cells with the TurboFect Transfection Reagent (Thermo Scientific, Waltham, MA, USA) in accordance with the manufacturer’s instructions. Briefly, the cells with 80% confluence were transfected using the Turbofect reagent in the DMEM medium and selected by puromycin (Invitrogen, San Diego, CA, USA) treatment (1μg/mL) for 4 days after transfection for 48 h. The cells were cultured supplementary with a complete medium until all cells survive.

The sg-HPGDS transfection efficiency was determined from the genomic sequence after cell collection. Briefly, the genomic DNA was extracted with the TIANamp Genomic DNA Kit (TIANGEN BIOTECH, Beijing, China). Genomic DNA was amplified with polymerase chain reaction (PCR), using the following primer (sense): ATACACAAAGAAACTAAGAACTGG, and antisense: ATTCTGTGTGTTCTCTATGCACC. The PCR product was sequenced and aligned (BGI, Chongqing, China). The sg-ACSL1 transfection efficiency was determined by Western blotting. To obtain HPGDS and ACSL1 double knockout cell lines, based on the sg-HPGDS cell lines, the sg-ACSL1 plasmids were transfected into the cells with the Transfection Reagent according to the manufacturer’s instructions.

### Transwell assay

To confirm the effect of HPGDS, ACSL1 on A549 cells migration, a total of 1×10^5^ sg-Con, sg-HPGDS, sg-ACSL1, sg-HPGDS and sg-ACSL1 double knockout cell lines diluted in DMEM without FBS were plated into the Transwell chamber (Corning), which was plated in a 24 well plate containing 600 μL of the complete medium. After 24 h, the cells that had migrated to the bottom of the membrane were fixed with 4% paraformaldehyde for 30 min, after which the membrane was stained with 0.1% crystal violet (Sangon Biotech, Shanghai, China). Eight fields were captured randomly under a microscope (Wetzlar, Germany). Crystal violet was eluted with 600 μL of 33% acetic acid, and the OD value was measured at 570 nm.

### Western blot analysis

Western blotting analysis was performed as described previously (27). Briefly, the cells were lysed with 1% SDS lysis buffer containing protease inhibitor cocktail and phosphatase inhibitor cocktail (Apexbio, Houston, USA). The protein concentration was determined by using a BCA protein assay reagent kit (Thermo Scientific). The protein content (18 μg) was separated by SDS-PAGE gels and transferred onto PVDF membranes (Millipore, Billerica, MA, USA). The PVDF membranes were blocked overnight by 5% fat free milk in TBST at 4°C, followed by incubation at 4°C for 12 h with primary antibodies for anti-β-actin (1:5000) (Sigma), anti-β-Tubulin (1:5000) (TransGen Biotech), anti-ACSL1 (1:1000) (Cell Signaling Technology), ACC (1:1000) (Cell Signaling Technology), HK2 (1:1000) (Cell Signaling Technology), ACAA1 (1:1000) (Cell Signaling Technology), E-cadherin (1:1000) (Bioworld), N-cadherin (1:1000) (Bioworld), Twist1 (1:1000) (Cell Signaling Technology), respectively. The membranes were washed with TBST and incubated for 1.5 h with the corresponding HRP-conjugated secondary antibodies. The bands were visualized with ECL reagents (Merck, Billerica, MA, USA). The western blots were analyzed with the Image Lab Software (BIO-RAD, USA), and the program included an application with protein gels; the image exposure time to was set to 16 s.

## Results

### Prognostic differential genes associated with lipid metabolism in lung adenocarcinoma

**Figure 1:**
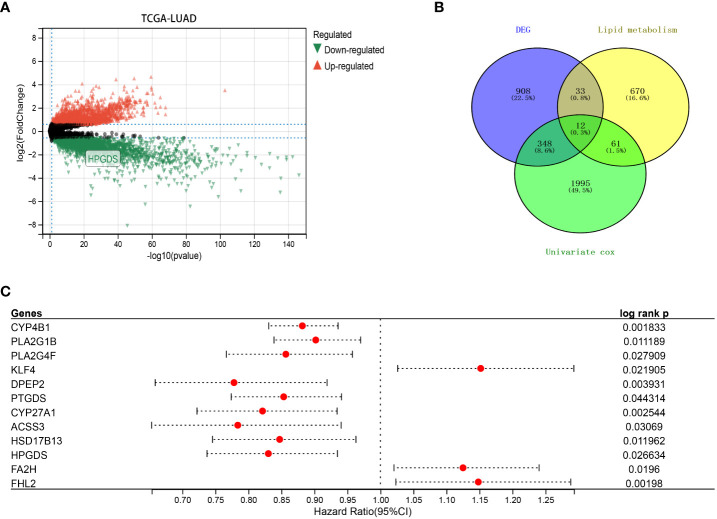
**(A)** Differentially expressed genes (DEGs) between LUAD and normal tissues in TCGA dataset. **(B)** Intersection of DEGs, lipid metabolism-related genes and prognostic genes in TCGA. **(C)** Univariate COX analysis of 12 key lipid metabolism genes (P<0.05).

Based on the TCGA dataset, we analyzed the differential genes of lung adenocarcinoma, among which 1301 genes showed differential expression, including 840 upregulated genes and 461 downregulated genes (**Figure 1A**). Univariate COX prognostic analysis of all genes identified 2416 genes with prognostic significance (**Figure 1C**; **Supplementary Table 1**). Following the method of Zheng et al. (24), we obtained a total of 776 lipid metabolism-related genes from the molecular signature database (version 7.0), KEGG and Reactome databases. After the further intersection with 1301 differential genes and 2416 prognostic genes, we finally found got 12 prognostic differences in lung adenocarcinoma expressed genes, and they were also associated with lipid metabolism (**Figure 1B**).

### Functional enrichment analysis

Further enrichment analysis of 45 genes showed that most of their biological processes were related to redox processes, lipid metabolism processes and lipoxygenase pathways, and were mainly located in the cytosol, endoplasmic reticulum membrane and lipid granules. It is also involved in iron ion binding, heme binding and oxidoreductase activity (**Supplementary Figure 1A**). The most relevant metabolic pathways are arachidonic acid metabolism and peroxisome proliferators-activated receptor (PPAR) signaling pathway (**Supplementary Figure 1B**). more over, 45 lipid metabolism-related differential genes were used to draw the PPI network map (**Supplementary Figure 1C**), and the top ten hub genes, (**Supplementary Figure 1D**) namely PPARG, ALOX15, ALOX5, PTGIS, PTGES, HPGDS, PLA2G1B, ALOX15B, CYP27A1, and CAV1 were visualized and calculated using Cytoscape.

### Construction of a prognostic risk score based on the TCGA cohort

**Figure 2:**
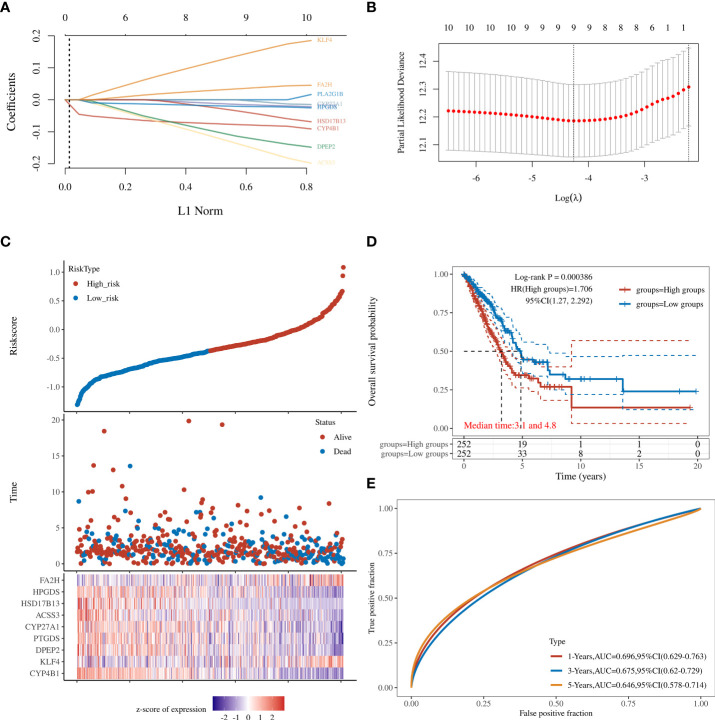
**(A)** LASSO regression analyses of the 12 OS-related genes. **(B)** Cross-validation for tuning the parameter selection in the LASSO regression. **(C)** The survival status for each patient (low-risk population: on the left side of the dotted line; high-risk population: on the right side of the dotted line). **(D)** Kaplan-Meier analysis for the OS of patients in the high- and low-risk groups. **(E)** The AUC of the prediction of 1-, 2-, and 3-year survival rates of LUAD.

Twelve genes were further analyzed by LASSO-Cox regression analysis. A 9-gene signature was constructed based on the optional λ value. The risk score is defined as Risk score = (−0.0797) *CYP4B1 + (0.1527) * KLF4 + (−0.1243) * DPEP2 + (−0.0165) * PTGDS + (−0.0057) * CYP27A1 + (−0.1551) * ACSS3 + (−0.0444) * HSD17B13 + (−0.0213) * HPGDS + (0.0381) * FA2H (**Figures 2A, B**).The TCGA cohort samples were divided into low- and high-risk groups according to the median cut-off value of the risk score (**Figure 2C**), and the Kaplan-Meier analysis showed that this risk grouping was effective in distinguishing between the good and poor prognosis groups. In other words, the OS of the low-score group in LUAD was statistically better than that of the high-score group (P<0.05) (**Figure 2D**). Time-dependent receiver operating characteristic (ROC) curves constructed to test the accuracy of the prognostic model, and the area under the curve (AUC) was 0.696 for 1 year, 0.675 for 3 years, and 0.646 for 5 years (**Figure 2E**). As shown in **Supplementary Figure 2**, these 12 genes have independent survival predictive power.

### Validation of prognostic models in the GEO cohort and gene set enrichment analyses

**Figure 3:**
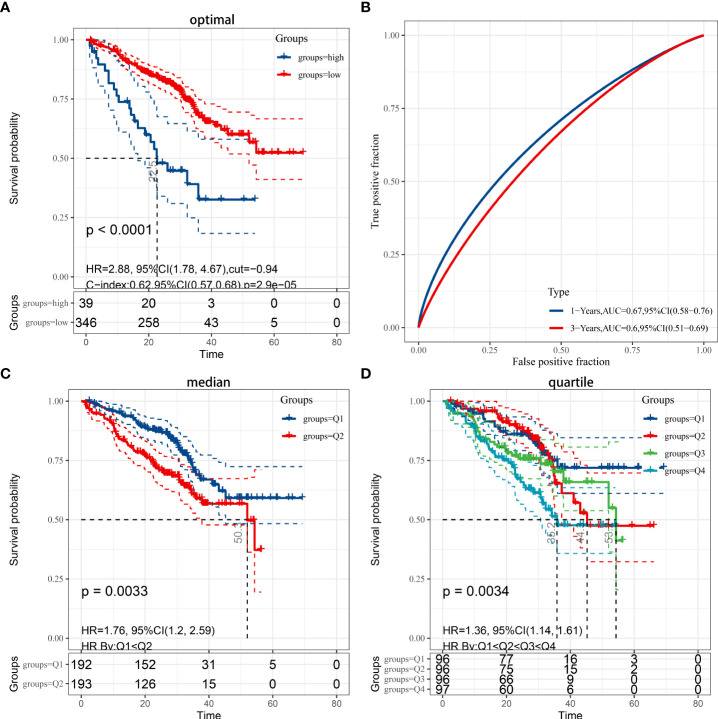
**(A)** Kaplan-Meier analysis applied for the evaluation of the risk scoring formula in the GSE72094 dataset, while considering the optimal cut-off value for grouping by P<0.05. **(B)** ROC curve in the validation set. **(C)** Grouped by median (P < 0.05). **(D)** Grouped by quartile (P < 0.05).

Patients with LUAD from the GEO dataset were involved in the validation of the risk model. Based on prognostic information from 442 patients enrolled in GSE72094, patients in the GEO cohort were divided into low- and high-risk groups based on the median risk score of the TCGA cohort, similar to the TCGA cohort. Kaplan-Meier analysis showed that overall survival was higher in the low-risk group than in the high-risk group (P < 0.01), regardless of whether the optimal cutoff (**Figure 3A**), median (**Figure 3C**), or quartile (**Figure 3D**). Due to incomplete 5-year survival data for patients in GSE72094, only an AUC of 0.0.67 at 1 year and 0.6 at 3 years was validated (**Figure 3B**).

To elucidate the underlying molecular mechanisms of risk scoring, we performed a GSEA comparison between high-risk and low-risk groups in the 442 patients in the data GSE72094. In the low-risk group, four oncological features of base excision repair, cell cycle, mismatch repair, and p53 signaling pathway, and a lipid metabolism feature of glyoxylate and dicarboxylate metabolism were enriched. In the high-risk group, the enriched KEGG pathway mainly focused on various cardiac disease associations (including dilated cardiomyopathy, virtual myocarditis, and cardiac muscle contraction, etc.). However, no significant enrichment was found in the oncological features (**Supplementary Figure 3**).

### Combining the TCGA-LUSC dataset to identify HPGDS as a key lipid metabolism gene

**Figure 4:**
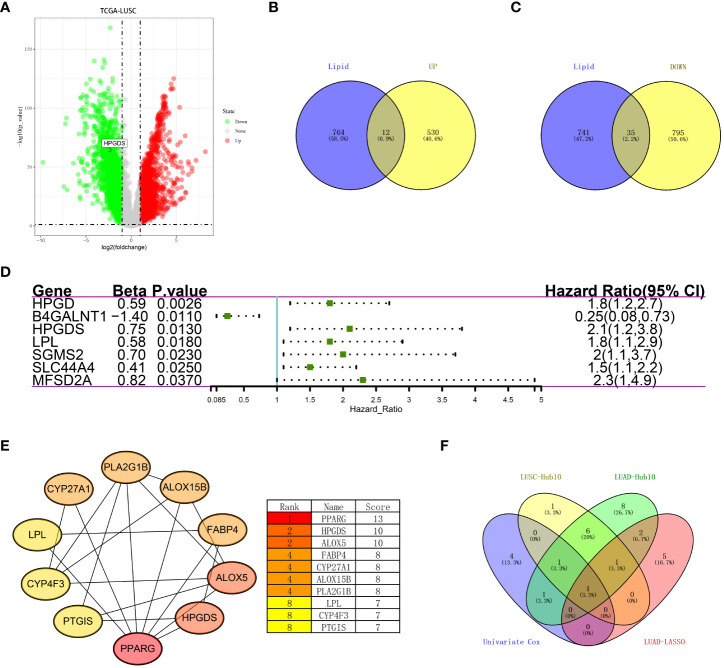
**(A)** Differentially expressed genes between LUSC and normal tissues in the TCGA dataset. **(B)** Upregulated lipid metabolism differential genes in tumor tissues. **(C)** Lipid metabolism differential genes downregulated in the tumor tissues. **(D)** Univariate COX analysis of key lipid metabolism genes (P<0.05). **(E)** Top10 hub genes were identified based on the PPI network map. **(F)** Recurrence of HPGDS in LUAD and LUSC.

As previously described, we performed differential analysis on TCGA data of lung squamous cell carcinoma (TCGA-LUSC) (**Figure 4A**; **Supplementary Figure 4A**) (LogFC = 2, FDR <0.05), and extracted genes related to lipid metabolism. A total of 542 upregulated genes and 830 downregulated genes was found in lung squamous cell carcinoma, including 12 and 35 genes related to lipid metabolism, respectively (**Figures 4B, C**). To further select the lipid metabolism-related genes with prognostic significance and to avoid the unreproducibility of a single dataset, we performed a univariate COX regression analysis on the above 47 important genes in the LUSC dataset GSE74777. The results showed that HPGD, B4GALNT1, HPGDS, LPL, SGMS2, SLC44A4, and MFSD2A were prognostically significant lipid metabolism-related genes (**Figure 4D**). Moreover, we also performed routine bioinformatics analysis of GO, KEGG and PPI for these 47 important genes (we consider count<20% as invalid enrichment). In terms of GO, they were enriched in two biological processes, oxidation-reduction process and lipid metabolic process, and were mainly located in the cytosol, extracellular exosome and endoplasmic reticulum membrane. However, related molecular functions were not enriched. Additionally, the results showed that the main pathway enrichments were metabolic pathways and arachidonic acid metabolism (**Supplementary Figure 4B**, **Supplementary Table 2**). Subsequently, we drew the PPI map of these 47 genes (**Supplementary Figure 4C**), and further analyzed the Hub genes among them using Cytoscape (v 3.8.0). The results showed that the top ten genes were PPARG, HPGDS, ALOX5, FABP4, CYP27A1, ALOX15B, PLA2G1B, LPL, CYP4F3 and PTGIS (**Figure 4E**). Notably, HPGDS simultaneously appeared in the HUB gene of the previous TCGA-LUAD dataset, the lasso regression prognostic model, the HUB gene of the TCGA-LUSC dataset, and the prognostic gene of LUSC (**Figure 4F**).

### Monogenic GSEA for HPGDS

**Figure 5:**
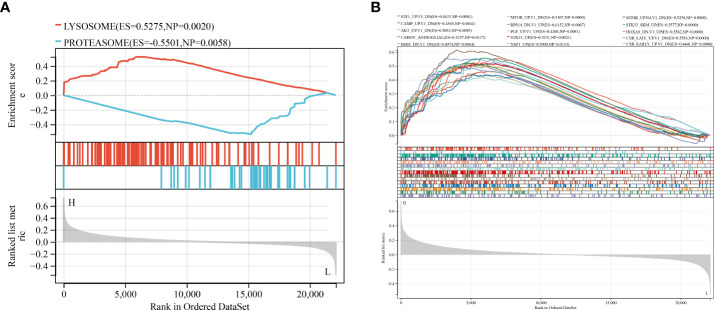
Single-gene GSEA analysis of HPGDS. **(A)** Enriched KEGG pathway. **(B)** Abundant tumor features.

To explore the underlying molecular mechanism of HPGDS, we performed a GSEA comparison between groups with different HPGDS expression. In terms of the KEGG pathway, the high expression group was enriched in lysosome, whereas proteasome was enriched in the low expression group (**Figure 5A**). Moreover, 15 oncological signatures including HOXA9, STK33, MTOR, RPS14, PGF, CSR, and YAP1 were enriched in the high expression group; however, no significantly enriched oncological signatures were found in the low expression group (**Figure 5B**). GO terms focused on ribosomal and mitochondrial function correlations. These enriched KEGG pathways and GO terms revealed molecular alterations in the HPGDS high expression group and were closely associated with metabolism. The results of GSEA are shown in **Supplementary Table 3**.

### Knockout of HPGDS is associated with lipid metabolism in lung adenocarcinoma

**Figure 6:**
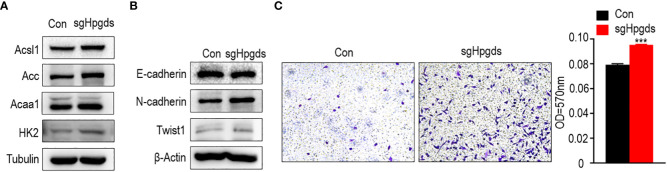
**(A)** Western blotting of the expression of lipid metabolism-related markers in normal control A549 cell line (NC) and HPGDS knockout A549 (sgHPGDS) group. **(B)**Knockdown of HPGDS promotes the EMT related markers of A549. **(C)** Knockdown of HPGDS enhances the migration of A549 cells. ***P<0.001.

Using CRISPR gene editing technology, we obtained the HPGDS knockout A549 cell line (Appendix 1). We examined differential expression of lipid metabolism-related proteins by western blotting. Compared with non-knockout cells, the expression of ACSL1, ACC, ACAA1, and HK2 was significantly increased after HPGDS knockout, which indicated that HPGDS knockout promoted lipid biosynthesis, and HPGDS can be associated with lipid metabolism (**Figure 6A**).

### Knockdown of HPGDS enhances lung adenocarcinoma migration

As shown in **Figure 6B**, the knockdown of HPGDS resulted in a decrease in E-cadherin and an increase in N-cadherin and TWIST1. Moreover, the migratory ability of A549 cells was examined using a transwell assay. A549 cells in the HPGDS knockout group were much higher than those in the control group (**Figure 6C**). These results INDICATED the role of HPGDS in the malignant progression of LUAD.

### Knockdown of HPGDS promoted migration by upregulating the expression of the lipid metabolism key enzyme ACSL1 and ACC

**Figure 7:**
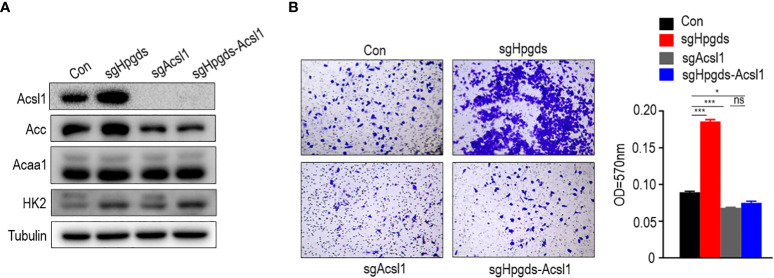
**(A, B)** Knockdown of HPGDS promoted migration by upregulate the expression of the lipid metabolism key enzyme ACSL1 and ACC, but not for HK2 and ACAA1. ns (no sense) P > 0.05, *P < 0.05, ***P < 0.001.

Rescue assays indicated that ACSL1 knockdown reversed the pro-migration effects of HPGDS knockdown. The knockdown of HPGDS promoted migration response by upregulating the expression of the lipid metabolism key enzymes ACSL1 and ACC (**Figure 7**).

## Discussion

The clinical efficacy of LUAD is not optimistic because owing to its extremely poor prognosis (28). Diagnostic biomarkers of LUAD and molecules should be identified for new therapeutic targets. Increased lipid uptake, storage, and lipogenesis occur in many cancers and increase tumor malignancy (29–31). Genes involved in lipid metabolism play a role in lipid metabolism reprogramming and drug resistance in tumors, making them potential targets for cancer therapy (32, 33). In this study, we aimed to discover potential biomarkers and therapeutic targets by identifying genes involved in lipid metabolism and their expression associated with prognosis in patients with LUAD, supplemented by biological experimental evidence.

Radiotherapy and chemotherapy for tumors have been developed vigorously in recent years. However, the 5-year survival rate of LUAD is still unsatisfactory (34–36). Risk score establishment based on bioinformatics analysis of RNA-sequencing data is an efficient approach, which classifies patients for rationally individualized and targeted treatment. Even though the risk models for the tumor microenvironment, immune cell infiltration, and energy metabolism of LUAD have been reported (37–39), we constructed a 9-gene prognostic risk model based on CYP4B1, KLF4, DPEP2, PTGDS, CYP27A1, ACSS3, HSD17B13, HPGDS, and FA2H, which was validated by an external dataset.

For a comprehensive understanding of the lipid metabolism process in lung cancer, we repeated the analysis pipeline of LUAD in the LUSC set, and in its results, the recurring appearance of HPGDS garnered our attention (**Supplementary Figures 1D**, **2A** and **Figure 4E**). Presently, tumor studies on HPGDS are limited, and many studies report them as tumor suppressor genes (22, 40). In recent years, most studies on tumors are related to bioinformatics; however, they lack experimental verification (22, 40–44). Therefore, we used CRISPR technology to knock out HPGDS in the A549 cell line. The detection of lipid metabolism pathways showed that the knockout of HPGDS promoted lipid synthesis. Besides, the knockdown of HPGDS promoted migration of A549 relative to the control group. The knockdown of HPGDS promoted migration response by upregulating the expression of the lipid metabolism key enzymes ACSL1 and ACC, but not for HK2 and ACAA1. Interestingly, the arachidonic acid metabolic pathway is an aberrant metabolic pathway in both LUAD and LUSC tissues. HPGDS happens to be involved in the arachidonic acid metabolic pathway as a σ class glutathione transferase (16).

Even though lipid metabolism pathways in lung cancer are widely studied (45–47), this study is different because it focused on the abnormal lipid metabolism pathways in patients with LUAD and established a more reliable prognostic risk score. We performed biological experiments and confirmed that HPGDS can promote the migration of A549 by upregulating the expression of key lipid metabolism enzymes ACSL1 and ACC, but not HK2 and ACAA1. This study will provide a good theoretical guide for further research on LUAD. However, the present study has some limitations. For example, the bioinformatics analysis of this study included lung squamous cell carcinoma; however, no biological experiments were performed, which will be a part of our follow-up work. In addition, there are insufficient biological experiments on HPGDS to completely explain how HPGDS leads to malignant changes in LUAD by affecting lipid metabolism pathways. Finally, our risk scoring formula also lacked validation on a large cohort.

To conclude, we investigated the abnormal lipid metabolism pathway of lung adenocarcinoma by bioinformatics and performed biological experiments to prove that HPGDS can lead to malignant changes by altering the lipid metabolism of lung adenocarcinoma. Therefore, the molecular mechanism underlying HPGDS regulating the lipid metabolism pathway in lung adenocarcinoma should be further studied.

## Data availability statement

The raw data supporting the conclusions of this article will be made available by the authors, without undue reservation.

## Author contributions

FS performed bioinformatics analyses and HM performed cell biology experiments. TL and QL were in-charge of data collection. LX and YX conceived and designed this article, and they were in charge of syntax modification and revised the article. All authors contributed to the article and approved the submitted version.

## Funding

This research was funded by Chongqing Science and Technology Commission Fund Project (grants number cstc2021jcyj-msxmX0312), National Natural Science Foundation of China (Grant No. 82030065, 81873932 and 81973567), Xi’an Municipal Health Commission Fund Project (grants number 2020yb04) and Xi’an Science and Technology Commission Fund Project (grants number 21YXYJ0032). The authors are thankful for all the fellows at the M.O.E. Key Laboratory of Laboratory Medical Diagnostics, the College of Laboratory Medicine, Chongqing Medical University.

## Acknowledgments

We are grateful to all the staff who contributed to the TCGA and GEO databases. Moreover, we are also thankful to the reviewers and editors for their sincere comments.

## Conflict of interest

The authors declare that the research was conducted in the absence of any commercial or financial relationships that could be construed as a potential conflict of interest.

## Publisher’s note

All claims expressed in this article are solely those of the authors and do not necessarily represent those of their affiliated organizations, or those of the publisher, the editors and the reviewers. Any product that may be evaluated in this article, or claim that may be made by its manufacturer, is not guaranteed or endorsed by the publisher.

## Abbreviations

LUAD, lung adenocarcinoma; GO, Gene Ontology; AUC, area under the curve; MF, molecular functions; ICIs, immune checkpoint inhibitors; ROC, receiver operating characteristic; GSEA, gene set enrichment analysis; KEGG, Kyoto Encyclopedia of Genes and Genomes; TCGA, The Cancer Genome Atlas; BP, biological processes; CC, cellular components; OS, overall survival; GEO, Gene Expression Omnibus; DEGs, differentially expressed genes.
