# Supplementary figures and images for: HPGDS is a novel prognostic marker associated with lipid metabolism and aggressiveness in lung adenocarcinoma
Source: Front Oncol. 2022 Oct 17;12:894485. doi: 10.3389/fonc.2022.894485 (PMC9618883; doi:10.3389/fonc.2022.894485)

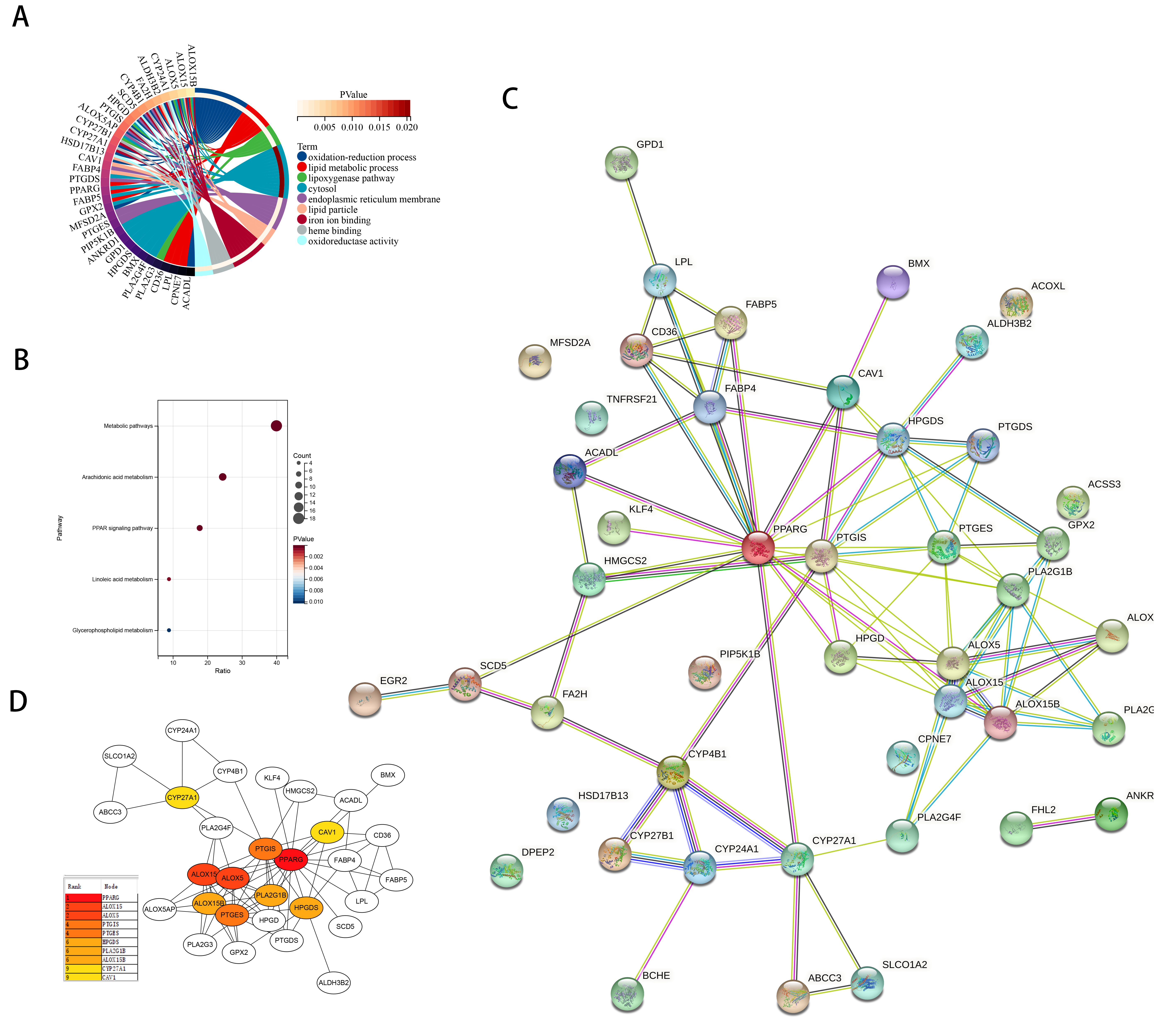

Supplement: Supplementary file 4 [file Image_1.tif]

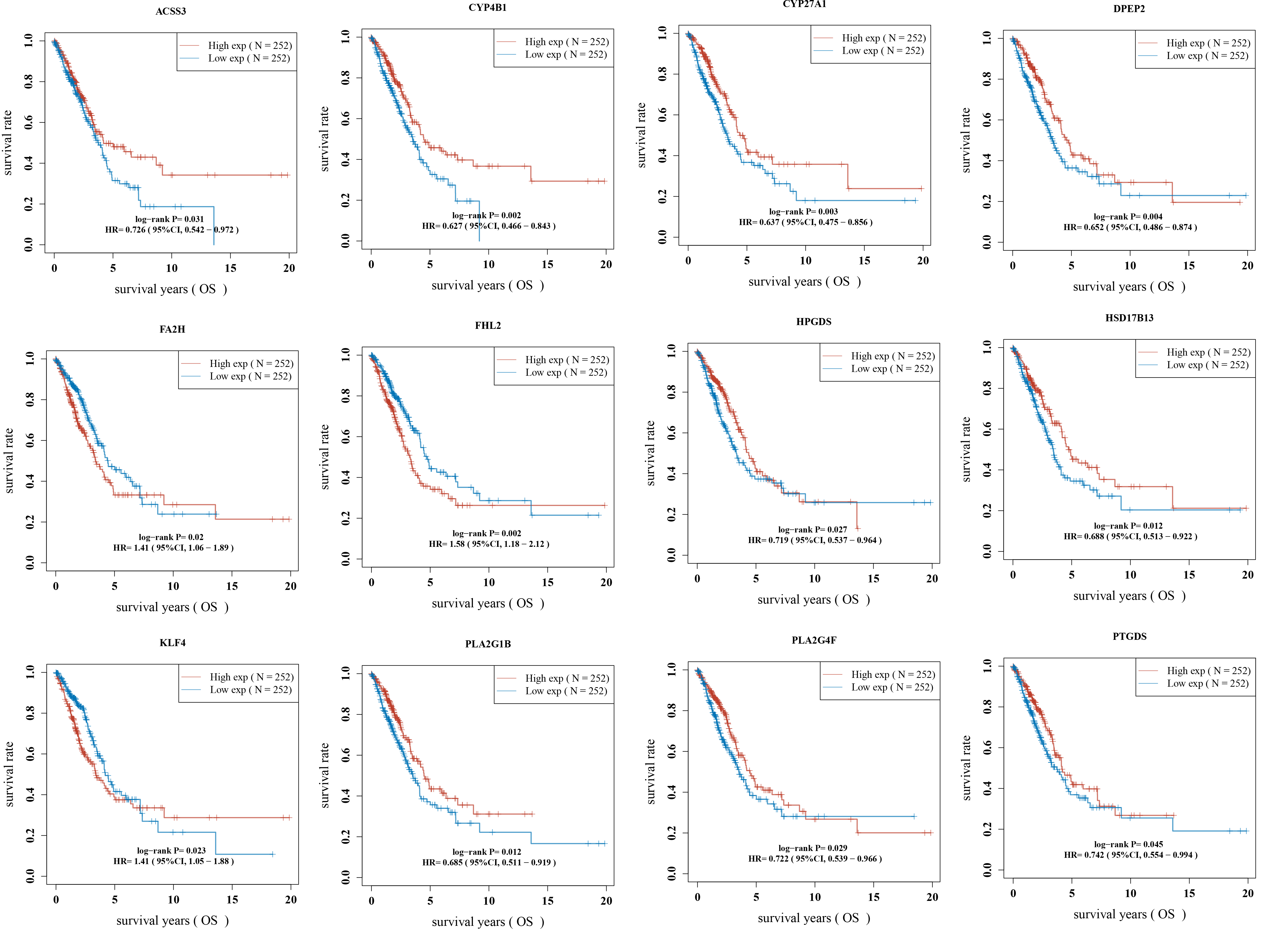

Supplement: Supplementary file 5 [file Image_2.tif]

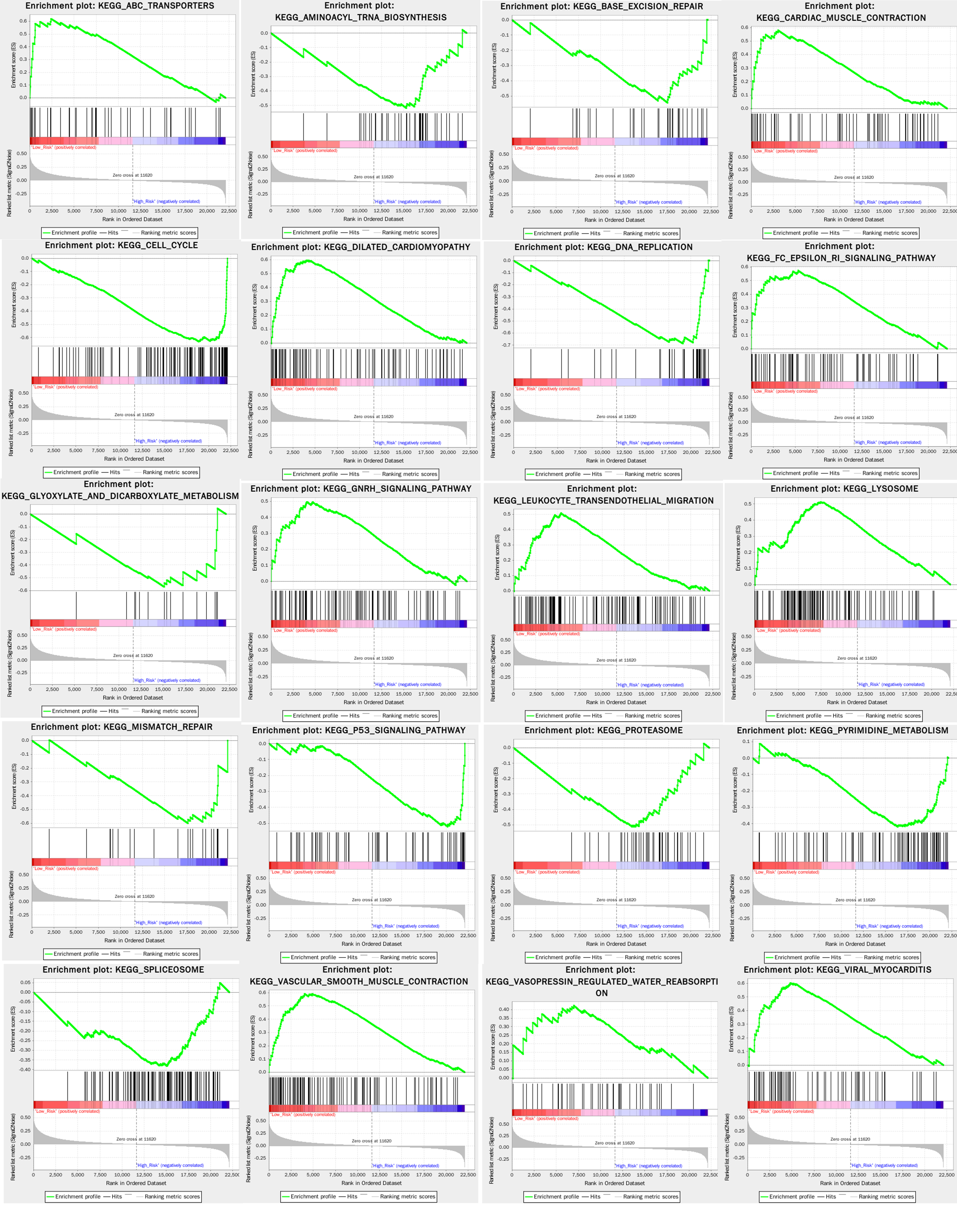

Supplement: Supplementary file 6 [file Image_3.tif]

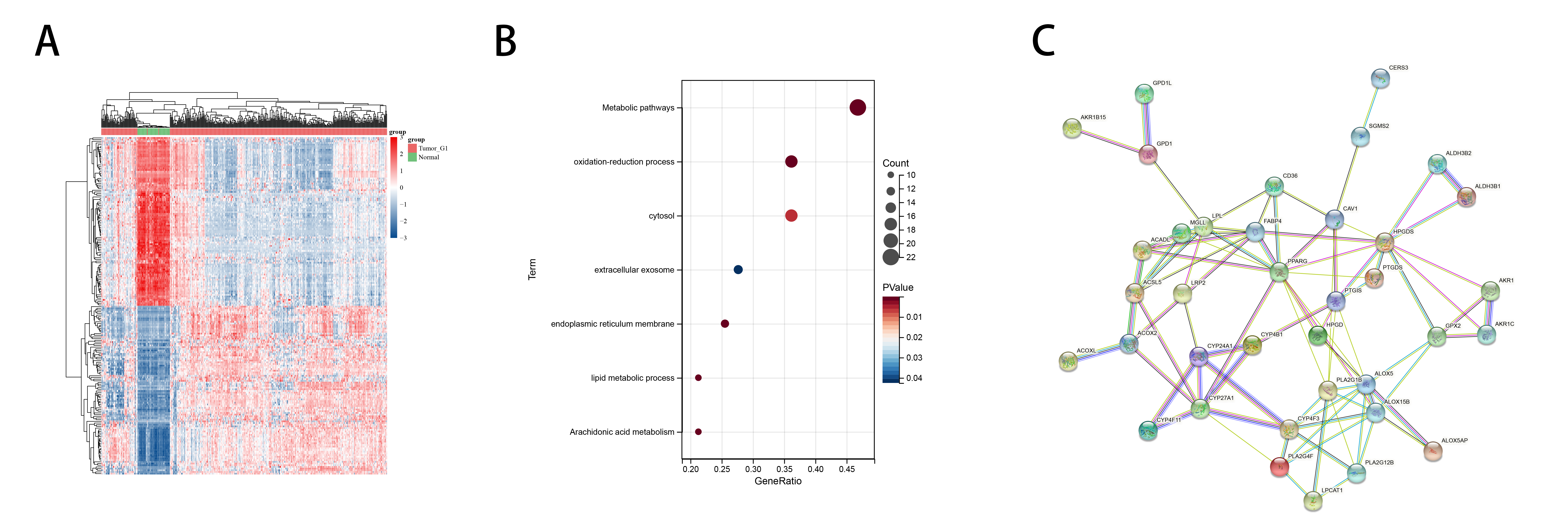

Supplement: Supplementary file 7 [file Image_4.tif]
